# Electron-Selective TiO_2_ Contact for Cu(In,Ga)Se_2_ Solar Cells

**DOI:** 10.1038/srep16028

**Published:** 2015-11-03

**Authors:** Weitse Hsu, Carolin M. Sutter-Fella, Mark Hettick, Lungteng Cheng, Shengwen Chan, Yunfeng Chen, Yuping Zeng, Maxwell Zheng, Hsin-Ping Wang, Chien-Chih Chiang, Ali Javey

**Affiliations:** 1 Electrical Engineering and Computer Sciences Department, University of California, Berkeley, CA 94720; 2 Materials Sciences Division, Lawrence Berkeley National Laboratory, Berkeley, CA 94720; 3 Green Energy & Environment Research Laboratorie, Industrial Technology Research Institute, 31040, Hsinchu, Taiwan, R.O.C

## Abstract

The non-toxic and wide bandgap material TiO_2_ is explored as an *n*-type buffer layer on *p*-type Cu(In,Ga)Se_2_ (CIGS) absorber layer for thin film solar cells. The amorphous TiO_2_ thin film deposited by atomic layer deposition process at low temperatures shows conformal coverage on the CIGS absorber layer. Solar cells from non-vacuum deposited CIGS absorbers with TiO_2_ buffer layer result in a high short-circuit current density of 38.9 mA/cm^2^ as compared to 36.9 mA/cm^2^ measured in the reference cell with CdS buffer layer, without compromising open-circuit voltage. The significant photocurrent gain, mainly in the UV part of the spectrum, can be attributed to the low parasitic absorption loss in the ultrathin TiO_2_ layer (~10 nm) with a larger bandgap of 3.4 eV compared to 2.4 eV of the traditionally used CdS. Overall the solar cell conversion efficiency was improved from 9.5% to 9.9% by substituting the CdS by TiO_2_ on an active cell area of 10.5 mm^2^. Optimized TiO_2_/CIGS solar cells show excellent long-term stability. The results imply that TiO_2_ is a promising buffer layer material for CIGS solar cells, avoiding the toxic CdS buffer layer with added performance advantage.

Among all thin film technologies, solar cells based on Cu(In,Ga)Se_2_ (CIGS) absorbers yield the best performance^1^. Recently, ZSW has reported its record CdS/CIGS solar cell with efficiency as high as 21.7%^2^. So far, CIGS cells with CdS buffer layer deposited by chemical bath deposition (CBD) have resulted in the highest conversion efficiencies. This is due to a suitable band alignment and *in-situ* surface passivation during CBD. The disadvantages of CdS, however, are parasitic absorption in the blue part of the solar spectrum and the toxicity of Cd^3^. Alternative buffer layers for CIGS which have been successfully employed are comprised of binary or multinary Zn(S,O,OH) and ZnMgO^4^,^5^,^6^,^7^. The aforementioned compounds have tunable band gaps larger than 3.5 eV and can form hole blocking contacts to CIGS^8^,^9^. Recently, ZSW presented a 21% efficient CIGS cell with zinc oxysulfide and zinc magnesium oxide layers substituting the conventional CdS buffer layer and ZnO front contact, respectively^10^,^11^. However, compound buffer layers consisting of Zn(S,O,OH) can have some disadvantages such as complicated reaction mechanism^12^, high resistivity (ZnS^13^), and light soaking effects^14^,^15^, presenting a potential cell reliability problem.

CIGS thin films deposited by vacuum processes such as co-evaporation and sputtering yield the highest efficiencies to date but require complex multistep processes^16^,^17^. Lower cost processing techniques would allow an important contribution to reduce the cost of photovoltaics. Non-vacuum deposition processes based on chemical precursor solution or nanoparticle printing offer the possibility to reduce the manufacturing costs, and give the flexibility for high throughput large area upscaling. An efficiency as high as 17.1% was demonstrated by printing a nanoparticle ink followed by rapid thermal processing to form CIGS^18^.

We chose non-vacuum deposited CIGS to demonstrate that TiO_2_ can be utilized as a selective electron contact on CIGS solar cells leading to a clear enhancement in photocurrent without compromising open-circuit voltage (V_oc_). Our results demonstrate that TiO_2_ is a promising candidate to successfully substitute the toxic CdS buffer layer. In this study, TiO_2_ thin films were deposited on printed nanoparticle based CIGS absorber layers by using the atomic layer deposition (ALD) technique. The ALD process provides good and uniform coverage as well as excellent thickness control^19^ on the surface of the CIGS absorber layer. To our knowledge, this is the first time that the wide band gap material TiO_2_ has been used successfully as alternative buffer layer in single junction planar CIGS solar cells.

## Results and Discussion

**Figure 1:**
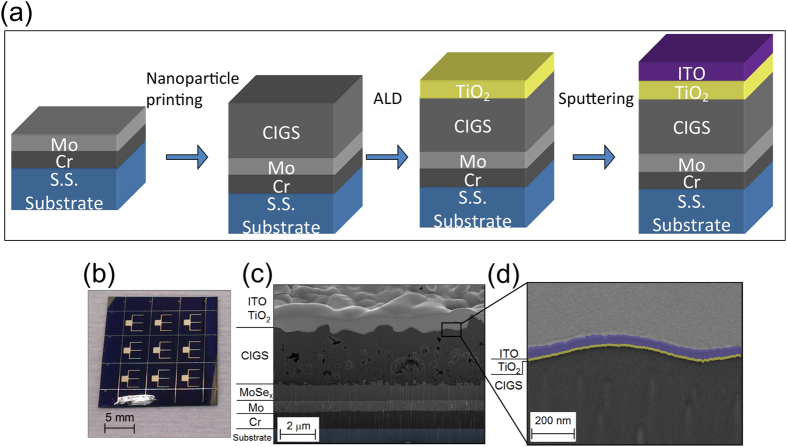
(**a**) Process schematic of the TiO_2_/CIGS solar cell, (**b**) photograph of the TiO_2_/CIGS solar cell device, (**c**) SEM cross-sectional view of a fully fabricated TiO_2_/CIGS solar cell prepared by FIB, (**d**) high resolution SEM cross-sectional view of ITO/TiO_2_/CIGS. The sample was coated with Pt to protect it during the FIB cut.

A sketch of the fabrication flow with the corresponding deposition methods is shown in Fig. 1(a). CIGS layers were deposited by non-vacuum printing of metal oxide powders on Cr/Mo coated stainless steel foil substrates (thickness 75 μm) followed by annealing in reducing H_2_ atmosphere and finally selenization in H_2_Se at 500 °C. As n-type buffer layer, either CdS or TiO_2_ is deposited by chemical bath deposition or ALD, respectively, followed by sputtering of the transparent electron contact indium tin oxide (ITO). The photograph in Fig. 1(b), shows a complete device with Ag grid fingers to facilitate electron extraction, manually scribed to cell sizes of 3.5 × 3.5 mm^2^, resulting in an active area of 10.5 mm^2^.

Figure 1(c) presents a focused ion beam prepared cross-sectional scanning electron microscope (SEM) image of the final TiO_2_/CIGS solar cell. Due to the reaction of Mo with H_2_Se, approximately 850 nm MoSe_2_ is formed at the interface of CIGS with Mo. The MoSe_2_ layer not only provides quasi-ohmic contact between Mo and CIGS but also builds up a backside field, which benefits hole transportation^20^. As typically observed in a two-step non-vacuum coating and selenization process, a bi-layer comprising a dense CIGS layer on the top and a porous nanocrystalline bottom layer forms. The dense upper layer is indium-rich (CuInSe_2_ (CIS)) while the bottom layer with small grains is gallium-rich (CuGaSe_2_) as measured by X-ray photoelectron spectroscopy (XPS) depth profiling (see supporting information Figure S1). The high magnification SEM image in Fig. 1(d) shows the conformal coverage of TiO_2_ on the CIGS absorber layer. The thickness of the amorphous TiO_2_ film was measured to be 15 nm.

**Figure 2:**
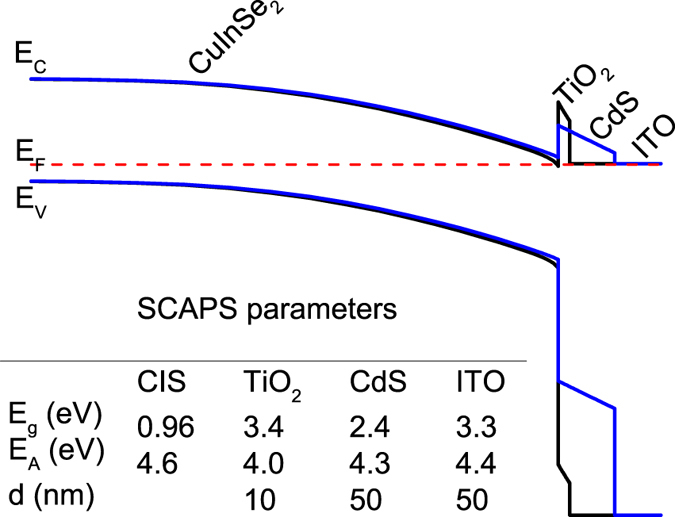
Schematic band diagrams of the CIS/TiO_2_/ITO (black line) and CIS/CdS/ITO (blue line) solar cells simulated with SCAPS. The table provides the basic input parameters for the simulation where E_C_, E_V_, E_F_, E_g_ and E_A_ are the conduction band minimum, valence band maximum, Fermi energy, band gap and electron affinity, respectively.

In our earlier work we characterized the structural and electronic properties of the TiO_2_ thin films deposited by ALD^21^. Atomic force microscopy and Raman spectroscopy revealed that TiO_2_ films grown at 120 °C are smooth and in an amorphous phase. XPS revealed close to stoichiometric TiO_2_ with a work function of 4.5 eV and the valence band maximum located at 7.4 eV. The band gap is 3.4 eV. In Fig. 2 we show a comparison of the schematic energy band diagrams of the ITO/TiO_2_/CIGS solar cell and the ITO/CdS/CIGS reference cell simulated by SCAPS under equilibrium conditions^22^. Please note, due to Ga segregation towards the back (compare supporting information, Figure S1), we simulate the buffer/absorber interface with CIS instead of CIGS. The relevant parameters for the energy band diagram simulation are listed in the table included in Fig. 2. The band diagram shows the p-CIS in contact with n-TiO_2_/ITO and n-CdS/ITO, respectively, to form the pn-junction. Both the TiO_2_ and CdS form a large barrier for majority carriers (holes) due to the large valence band offset with the CIS absorber. The positive conduction band offset (spike) for the TiO_2_/CIS interface is larger than for the CdS/CIS reference cell due to a lower electron affinity of TiO_2_ (4.0 eV^21^) compared to CdS (4.3 eV^23^). The SCAPS simulation of the TiO_2_/CIGS sample hypothesizes an inverted surface which might help to reduce recombination at the TiO_2_/CIGS interface. Further experiments are needed to verify this simulation.

**Figure 3:**
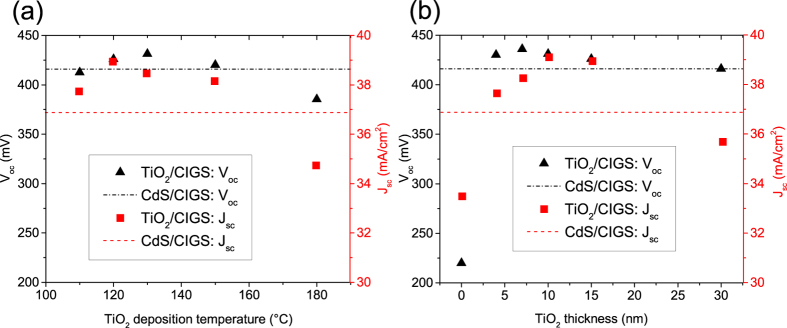
(**a**) Dependence of V_oc_ and J_sc_ on TiO_2_ deposition temperature (at fixed TiO_2_ thickness of 15 nm), and (**b**) dependence on TiO_2_ thickness (at fixed deposition temperature of 120 °C). The V_oc_ and J_sc_ of the CdS reference cell are given as dashed lines.

To optimize the device performance, the influence of the TiO_2_ deposition temperature and thickness on V_oc_ and short-circuit current density (J_sc_) were investigated (Fig. 3(a,b), respectively). The parameters obtained under optimized conditions for the CdS reference cell are given as dashed lines. When increasing the TiO_2_ deposition temperature, the thickness was fixed to 15 nm. As shown in Fig. 3(a), the V_oc_ of the TiO_2_/CIGS solar cells rises from 412 mV to 431 mV as the deposition temperature increases from 110 °C to 130 °C, which is above the V_oc_ of the CdS/CIGS reference cell (416 mV). A possible reason for the V_oc_ enhancement might be elemental interdiffusion at the TiO_2_/CIGS interface which could lead to the formation of an inverted surface accompanied by a drop in interface recombination. The J_sc_ of the TiO_2_/CIGS solar cells reaches its maximum of 38.9 mA/cm^2^ at a TiO_2_ deposition temperature of 120 °C. A further temperature increase leads to both V_oc_ and J_sc_ drop to as low as 385 mV and 34.7 mA/cm^2^ at 180 °C, respectively. As it was found earlier by Yin *et al.*^21^ the TiO_2_ film deposited at 120 °C is in an amorphous phase and shows a smooth morphology. As the temperature increases the phase changes from amorphous to nanocrystalline accompanied by a rougher morphology^21^, which possibly explains the drop in all photovoltaic parameters. However, at elevated deposition temperature not only the phase of the TiO_2_ might play an important role for the pn-junction formation but also possible ion diffusion at the TiO_2_/CIGS interface or within the CIGS which should be the subject of further studies.

To study the influence of the TiO_2_ thickness, the deposition temperature was fixed at 120 °C (Fig. 3(b)). The V_oc_ and J_sc_ of TiO_2_/CIGS solar cells increase with TiO_2_ thickness up to 10 nm. A maximum V_oc_ of 436 mV and J_sc_ of 39.1 mA/cm^2^ are measured for 7 and 10 nm TiO_2_, respectively. As the TiO_2_ thickness exceeds 15 nm the J_sc_ drops significantly due to residual light absorption while the V_oc_ remains at the CdS/CIGS reference level.

**Figure 4:**
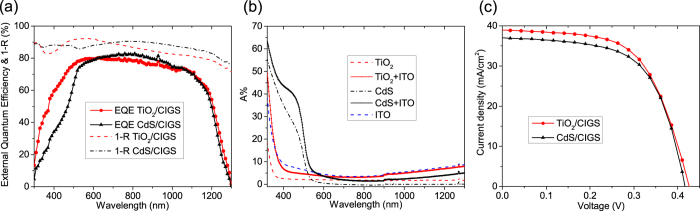
(**a**) EQE and 1-Reflectance curves of the TiO_2_/CIGS and CdS/CIGS solar cells, (**b**) absorption curves of TiO_2_, CdS, ITO, TiO_2_/ITO, and CdS/ITO. (**c**) J-V curves of TiO_2_/CIGS and CdS/CIGS solar cells corresponding to the EQE data shown in (**a**).

Inspection of the external quantum efficiency (EQE) curves of the TiO_2_/CIGS and CdS/CIGS devices in Fig. 4(a) reveal a significant photocurrent gain in the wavelength range between 300 and 630 nm. Between 630 and 1060 nm the EQE of the CdS/CIGS reference device is slightly higher, this however can be correlated to a reduced light in-coupling caused by higher reflectance losses (R, presented as 1-R) in the TiO_2_/CIGS device seen in Fig. 4(a). Higher reflectance losses in the TiO_2_/CIGS solar cell might be caused by a slightly non-ideal total oxide thickness that allows for constructive interference of the reflected light but can be circumvented by applying an anti-reflection coating. The minimum band gaps of the CIGS absorbers are 0.95 eV (with TiO_2_) and 0.96 eV (with CdS) as extracted from the sharp EQE cut-off at high wavelengths. In Fig. 4(b) we show the absorption (A%) of the individual window layers TiO_2_ (10 nm), CdS (50 nm) and ITO (50 nm) as well as their combinations TiO_2_/ITO and CdS/ITO deposited on quartz glass substrates. Both, the absorption onsets of TiO_2_ and CdS match well with their band gaps of 3.4 eV and 2.4 eV, respectively. The CdS/ITO bilayer shows high absorption for wavelengths <600 nm as compared to the TiO_2_/ITO bilayer accounting for the significant gain in photocurrent for the latter device stack as previously discussed.

The current-voltage (J-V) characteristics of the optimized TiO_2_/CIGS device and a reference CdS/CIGS device are shown in Fig. 4(c) (corresponding cell parameters are summarized in Table 1). The best performance, mainly due to a significant photocurrent gain of 2.0 mA/cm^2^ was obtained with 10 nm amorphous TiO_2_ deposited at 120 °C. The gain in photocurrent seen in the TiO_2_/CIGS device can be even higher by optimizing the antireflection coating as indicated by the 1-R measurements (Fig. 4(a)). The fill factor (FF) of the reference CdS/CIGS solar cell is slightly higher than the FF of the TiO_2_/CIGS cell, which are 61.4% and 59.6%, respectively. This difference is caused by the higher series resistance of 2.8 Ohm cm^2^ (TiO_2_/CIGS) as compared to 1.9 Ohm cm^2^ (CdS/CIGS) probably related to a larger resistivity of the TiO_2_ thin film. This could be mitigated in the future by doping the TiO_2_ layer. It is assumed that the porous CIGS absorber (compare Fig. 1(c)) gives rise to the relatively high series resistance observed in all devices. The V_oc_ of the TiO_2_/CIGS solar cell (426 mV) does not suffer from a higher conduction band offset presented in the simulated band diagram (Fig. 2) and is marginaly higher than the 416 mV measured in the CdS/CIGS solar cell. Finally, the TiO_2_/CIGS device shows an active area efficiency of 9.9%, which is slightly better than the efficiency of the CdS/CIGS reference device (9.5%).

To asses the long-term stability of the TiO_2_/CIGS (15 nm TiO_2_ deposited at 120 °C) and CdS/CIGS solar cells, J-V curves were remeasured after 9 months and are presented in Fig. S2 (supporting information) and are summarized in Table 1. Both devices show degradation over time. The TiO_2_/CIGS solar cell only suffers FF degradation which fully recovers under light soaking within 20 minutes resulting in a slightly improved cell efficiency due to a marginal increase in short circuit current. The CdS/CIGS reference solar cell degrades in FF as well as short circuit current density which do not recover under light soaking leading to an efficiency drop (Table 1).

In conclusion, the wide bandgap and non-toxic material TiO_2_ deposited by ALD was successfully used as the n-type buffer layer for non-vacuum deposited CIGS thin film solar cells on flexible stainless steel substrates. A J_sc_ gain of 2.0 mA/cm^2^ was achieved by substitution of the conventional CdS buffer layer, resulting in a photocurrent of 38.9 mA/cm^2^. The ultrathin TiO_2_ layer dramatically enhanced the photocurrent gain in the UV spectrum without compromising V_oc_ due to its homogeneous and conformal coverage, inherent to the ALD deposition process, and possibly a surface inversion at the TiO_2_/CIGS interface. We conclude that the ultrathin amorphous TiO_2_ layer is a promising candidate for the application in high efficiency CIGS thin film solar cells to further boost their performance.

## Methods

### Cu(In,Ga)Se_2_ preparation

Metallic oxide powders of In_2_O_3_, Ga_2_O_3_ and Cu_2_O were mixed homogeneously with DI water and subsequently coated onto the Mo/Cr substrate by using a doctor blade. The precursor was converted to CIGS first by reduction in hydrogen (H_2_) replace: followed by selenization for 20 minutes in hydrogen selenide (5% H_2_Se in Ar), both at a temperature of 500 °C.

### Solar cell device fabrication

The following device architecture was employed: stainless steel foil/Cr/Mo/CIGS/CdS or TiO_2_/ITO/Ag. 1000 nm Cr and 800 nm Mo were deposited on 75 μm stainless steel foil substrate by DC sputtering used as diffusion barrier and back contact, respectively. Before depositing the n-type buffer layer, the CIGS absorber layers were rinsed in 5 wt% potassium cyanide solution for 5 minutes to remove excess CuSe_x_. Next, the CIGS absorbers were coated with TiO_2_ by atomic layer deposition (ALD). TiO_2_ thicknesses varied from 0–30 nm and deposition temperatures ranged from 110–180 °C. Titanium isopropoxide (Ti[OCH(CH_3_)_2_]_4_) and H_2_O were used as ALD precursors. The TiO_2_ deposition rate is about 0.25 Å/s measured by ellipsometry on test films grown on Si wafer substrates. A reference sample was immersed under optimized conditions^24^,^25^ in a chemical bath to deposit 50 nm CdS at a temperature of 70 °C (details can be found in ref. 24). 50 nm indium tin oxide (ITO) as transparent conductive layer was deposited on both CdS/CIGS and TiO_2_/CIGS devices by RF sputtering at room temperature (ITO target: In_2_O_3_:SnO_2_ = 90%:10%). ITO was deposited by a mild off-angle sputtering condition using 40 Watt and 0.9 mTorr Ar pressure with a sample-to-target distance of 17 cm. Finally, shadow masks were used to define the metal grid patterns. 100 nm thick Ag (thermally evaporated) grid with finger width and pitch of 100 μm and 800 μm, respectively, were deposited by thermal evaporation. Individual cells of 3.5 × 3.5 mm^2^ size were manually scribed resulting in an active area of 10.5 mm^2^.

### Focused ion beam preparation

The sample was coated with Pt to prevent damage during the FIB cut. FIB was done on a FEI Quanta 3D FEG with Ga ions using 30 kV and 50 pA.

### Scanning electron microscope

Cross sectional scanning electron micrographs were taken on a Zeiss Gemini Ultra-55.

### Simulation

The 1D solar cell simulation software SCAPS^22^ was used to simulate the CIS/CdS/ITO and CIS/TiO_2_ /ITO interface. The band gaps and electron affinities of 0.96 eV and 4.6 eV (CIS), 3.4 eV and 4.0 eV (TiO_2_), 2.4 eV and 4.3 eV (CdS) and 3.3 eV and 4.4 eV (ITO) were used for the simulations.

### Solar cell performance measurement: J-V, EQE and 1-R%

Solar cells were characterized under simulated 1-sun illumination (1000 W/m^2^, global air mass 1.5 spectrum, 25 °C). EQE was measured in a QE-R system from Enlitech using a 150W XQ lamp. Calibrated Si (300 nm–1100 nm) and a Ge (1100 nm–1800 nm) diodes were used as references for the EQE measurement. Barium sulfate was used as calibration sample to represent 100% reflectance.

### Transmission and reflection measurements

Done with a Lambda 950 UV/VIS Spectrometer from PerkinElmer using a tungsten lamp. A% was extracted from reflectance R% and transmission T% spectroscopy via A% =100 % – R% – T%, where R% and T% are corrected for the quartz glass substrate.

## Additional Information

**How to cite this article**: Hsu, W. *et al.* Electron-Selective TiO_2_ Contact for Cu(In,Ga)Se_2_ Solar Cells. *Sci. Rep.* **5**, 16028; doi: 10.1038/srep16028 (2015).

## Supplementary Material

Supporting Information

## Figures and Tables

**Table 1:** V_oc_, J_sc_, FF and efficiency of the best TiO_2_/CIGS solar cell as well as the CdS/CIGS reference solar cell alongside with stability measurements of a TiO_2_/CIGS (15 nm TiO_2_ deposited at 120 °C) and CdS/CIGS solar cell re-measured after 9 months and under light soaking.

	Configuration	V_oc_ (mV)	J_sc_ (mA/cm^2^)	FF (%)	Eff. (%)
Best	CdS/CIGS	416	36.9	61.4	9.5
Stability test, first measured	CdS/CIGS	401	38.2	55.8	8.5
After 9 months	CdS/CIGS	396	36.2	52.5	7.5
After 9 months + light soak	CdS/CIGS	395	36.1	53.2	7.6
Best	TiO_2_/CIGS	426	38.9	59.6	9.9
Stability test, first measured	TiO_2_/CIGS	404	39.8	56.4	9.1
After 9 months	TiO_2_/CIGS	399	40.0	50.7	8.1
After 9 months + light soak	TiO_2_/CIGS	404	40.2	56.5	9.2

Individual cells are scribed into areas of 3.5 × 3.5 mm^2^, resulting in an active area of 10.5 mm^2^.
