# Supplementary material for: Electron-Selective TiO2 Contact for Cu(In,Ga)Se2 Solar Cells
Source: Sci Rep. 2015 Nov 3;5:16028. doi: 10.1038/srep16028 (PMC4630620; doi:10.1038/srep16028)
Supplement: Supporting Information [file srep16028-s1.pdf]

Supporting information

Electron-Selective TiO_2 Contact for $Cu(In,Ga)Se_2$ Solar Cells

Weitse Hsu[1,2,3▽], Carolin M. Sutter-Fella[1,2▽], Mark Hettick[1,2], Lungteng Cheng[3], Shengwen Chan[3], Yunfeng Chen[3], Yuping Zeng[1], Maxwell Zheng[1,2], Hsin-Ping Wang[1,2], Chien-Chih Chiang[3], and Ali Javey[1,2,*]

[1]*Electrical Engineering and Computer Sciences Department, University of California, Berkeley, CA 94720*

[2]*Materials Sciences Division, Lawrence Berkeley National Laboratory, Berkeley, CA 94720*

[3]*Green Energy & Environment Research Laboratorie, Industrial Technology Research Institute, 31040, Hsinchu, Taiwan, R.O.C.*

*Corresponding Author: ajavey@eecs.berkeley.edu

[▽]These authors contributed equally to this work

Elemental depth profiles

Fig. S1a shows a slow XPS sputter depth profile (sputter rate ~1.3 A/s based on a SiO_2 reference sample) of the ITO/TiO_2 and CIGS surface, which clearly confirms the presence of the thin TiO_2 interlayer (15 nm). Fig. S1b with a faster sputter rate depicts the depth profile of the complete ITO/TiO_2/CIGS/Mo stack. No Ga was detected at the surface but only segregated to the bottom of the CIGS layer. Moreover, the Se signal does not drop at the CIGS/Mo interface due to the formation of $MoSe_2$ during the selenization process.

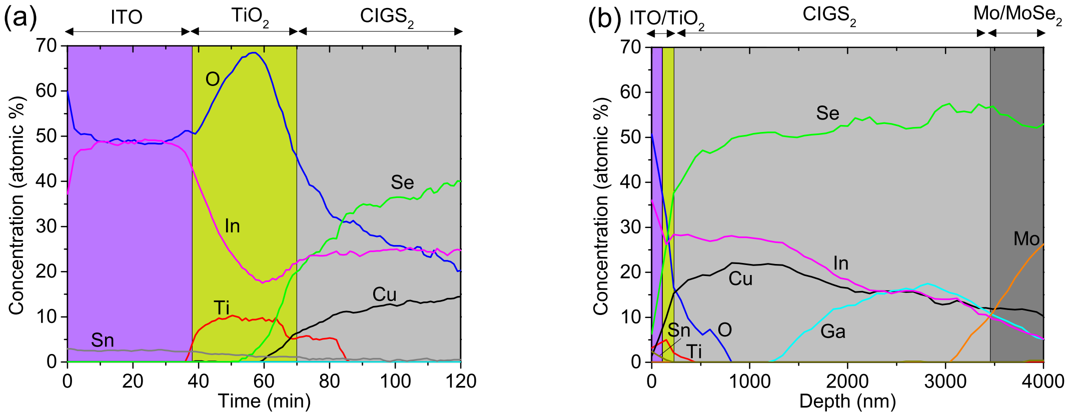

Figure S1. Elemental XPS depth profiles. (a) slow sputtering of ITO/TiO_2 and CIGS surface, (b) full ITO/TiO_2/CIGS/Mo depth profile.

Solar cell long term stability

To asses the long-term stability of the TiO_2/CIGS (with 15 nm TiO_2 deposited at 120°C) and CdS/CIGS solar cells, J-V curves were remeasured after 9 months under 1 sun illumination and are presented in Fig. S2. Both devices show degradation over time. The TiO_2/CIGS solar cell only suffers FF degradation (see Table 1) which fully recovers under light soaking within 20 minutes resulting in a slightly improved cell efficiency due to a marginal increase in short-circuit current. The CdS/CIGS reference solar cell degrades in FF as well as short-circuit current density which do not recover under light soaking leading to an efficiency drop (Table 1).

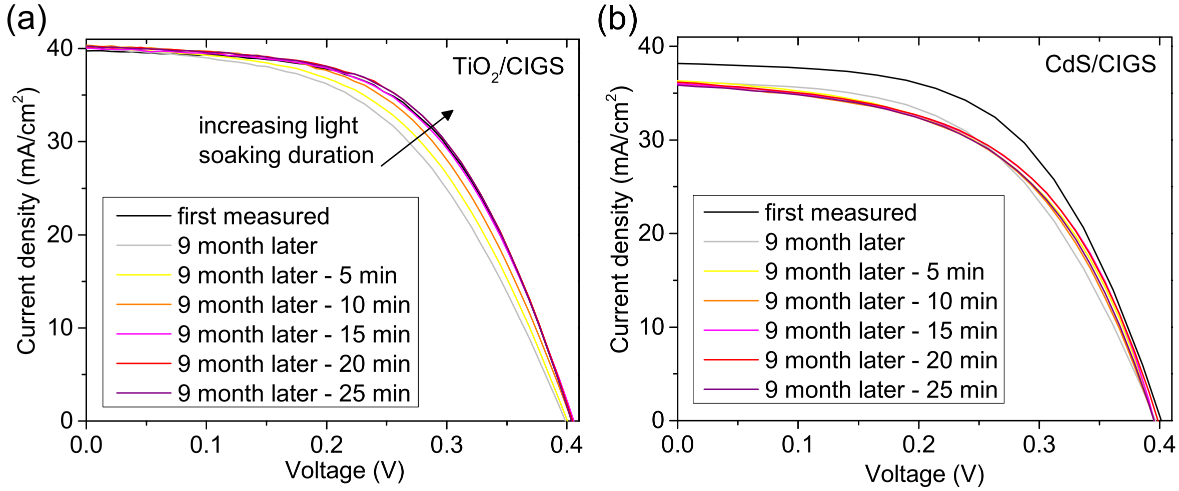

Figure S2. J-V curves of (a) TiO_2/CIGS (with 15 nm TiO_2) and (b) CdS/CIGS solar cells as it was first measured and 9 months later under 1 sun light soaking for up to 25 minutes.

Methods

Elemental depth profile. X-ray photoelectron spectroscopy (XPS) of a full ITO/TiO_2/CIGS/Mo stack was performed in a Thermo Scientific K-Alpha instrument from Physical Electronics. A monochromatic Al Kα radiation (1486.7 eV) with a X-ray spot size of 400 μm were used as excitation source. The slow depth profile (Fig. S1a) was obtained by using an argon ion gun at an energy of 1 keV with a raster size of 2 mm. The full depth profile with faster sputter rate (Fig. S1b) was obtained by using an argon ion gun at an energy of 3 keV and a raster size of 1.25 mm.

Solar cell stability. TiO_2/CIGS and CdS/CIGS solar cells were remeasured after 9 months under simulated 1-sun illumination (1000 W/m^2, global air mass 1.5 spectrum, 25°C). If light soaking was applied, the cells were left under 1-sun illumination for the given amount of time before J-V data were acquired.
